# Mammary epithelium‐specific inactivation of V‐ATPase reduces stiffness of extracellular matrix and enhances metastasis of breast cancer

**DOI:** 10.1002/1878-0261.12159

**Published:** 2017-12-12

**Authors:** Gajendra K. Katara, Arpita Kulshrestha, Liqun Mao, Xin Wang, Manoranjan Sahoo, Safaa Ibrahim, Sahithi Pamarthy, Kimiko Suzue, Gajendra S. Shekhawat, Alice Gilman‐Sachs, Kenneth D. Beaman

**Affiliations:** ^1^ Department of Microbiology and Immunology Chicago Medical School Rosalind Franklin University of Medicine and Science North Chicago IL USA; ^2^ Department of Materials Science and Engineering Northwestern University Evanston IL USA; ^3^ Department of Pathology Chicago Medical School Rosalind Franklin University of Medicine and Science North Chicago IL USA; ^4^ Advocate Lutheran General Hospital Park Ridge IL USA

**Keywords:** atomic force microscopy, *ATP6V0a2*, breast cancer metastasis, extracellular matrix stiffness, glycosylation, V‐ATPase

## Abstract

Extracellular matrix (ECM) critically impacts tumor progression and is influenced by both cancer and host tissue cells. While our understanding of cancer cell ECM remodeling is widespread, the importance of host tissue ECM, which provides initial congenial environment for primary tumor formation, is partly understood. Here, we report a novel role of epithelial cell‐associated vacuolar ATPase ‘a2’ isoform (a2V) in regulating breast tissue ECM stiffness to control metastasis. Using a mammary gland‐specific a2V‐knockout model, we show that in the absence of a2V, breast tumors exhibit atypically soft tumor phenotype, less tumor rigidity, and necrotic tumor microenvironment. These tumors contain a decreased number of cancer cells at primary tumor site, but showed extensive metastases compared to control. Nanomechanical evaluation of normal breast tissues revealed a decrease in stiffness and collagen content in ECM of a2V‐deleted breast tissues. Mechanistically, inhibition of a2V expression caused dispersed Golgi morphology with relocation of glycosyltransferase enzymes to early endosomes in mammary epithelial cells. This resulted in defective glycosylation of ECM proteins and production of compromised ECM that further influenced tumor metastasis. Clinically, in patients with cancer, low a2V expression levels in normal breast tissue correlated with lymph node metastasis. Thus, using a new knockout mouse model, we have identified a2V expression in epithelial cells as a key requirement for proper ECM formation in breast tissue and its expression levels can significantly modulate breast tumor dissemination. Evaluation of a2V expression in normal breast tissues can help in identifying patients with high risk of developing metastases.

**Abbreviations**
(a2V^fl/fl^): floxed *ATP6v0a2* gene
a2V: ‘a2’ subunit isoform of V0 domain of V‐ATPase
ACRL: autosomal recessive cutis laxa
AFM: atomic force microscopy
Cre: Cre recombinase
ECM: extracellular matrix
GL25D2: galactosyltransferase
HMEpC: human mammary primary epithelial cells
LNM: lymph node metastasis
MMTV: mouse mammary tumor virus
PNA: peanut agglutinin
SNA: *Sambucus nigra* agglutinin
V‐ATPase: vacuolar ATPase

## 1 Introduction

The local microenvironment or niche around tumors plays a significant role in initiating and encouraging tumor invasion and metastasis. A successful metastasis requires a local niche to support cancer cell proliferation and formation of a primary tumor. This niche contains blood cells, immune cells, fibroblasts, endothelial cells, and extracellular matrix (ECM) (Bonnans *et al*., 2014; Liotta and Kohn, 2001). ECM is a complex network of macromolecules with distinct biological and mechanical properties and regulates the cellular behavior directly or indirectly (Lu *et al*., 2011). In cancer, ECM architecture can influence disease outcome before and during the disease conditions (Lu *et al*., 2012). For example, overexpression of hyaluronan synthase‐2 causes high molecular mass hyaluronan production in ECM that protects against malignant tumor progression (Tian *et al*., 2013). During cancer, ECM remodeling occurs through rearrangement, cross‐linking, and deposition as well as degradation of certain ECM proteins (Paszek *et al*., 2005). This remodeling of ECM has been suggested as a pathway rather than an obstacle for cancer metastasis (Lu *et al*., 2011, 2012). Nevertheless, the molecules that can influence ECM organization in normal condition can be of immense importance in cancer prognosis or therapeutics.

Vacuolar (V)‐ATPases are the multi‐subunit proton pumps present on vesicular membranes of normal cells and maintain intracellular pH. In cancer cells, V‐ATPases are required on the cell surface where they actively participate in growth, metastasis, and chemoresistance (Forgac, 2007; Kulshrestha *et al*., 2015, 2016; Smith *et al*., 2016; Stransky *et al*., 2016). Several V‐ATPase subunits have multiple isoforms that exhibit cell‐ and tissue‐specific expression, thereby contributing to a variety of physiological activities within the cell (Breton and Brown, 2013; Smith *et al*., 2003). In humans, mutations in the *ATP6V0a2* gene, which encodes V‐ATPase ‘a2’ isoform (a2V), lead to glycosylation defects of serum proteins and cause the autosomal recessive cutis laxa (ACRL) skin syndrome (Guillard *et al*., 2009; Kornak *et al*., 2008). Inhibition of a2V expression in mammary epithelial cells causes impairment in cell proliferation through Notch and TGF signaling (Pamarthy *et al*., 2016).

In cancer, we have reported the role of cancer cell‐associated a2V in promoting protumorigenic macrophage phenotype in the breast tumor microenvironment (Katara *et al*., 2014, 2016). However, the precise role of V‐ATPase in host tissue microenvironment during breast cancer dissemination is not clear. In this study, using an a2V‐knockout mouse model, we show that the inhibition of V‐ATPase expression in mammary epithelial cells modulates the ECM architecture that directly affects breast cancer phenotype and metastasis in transplant tumor model. We demonstrate that deletion of a2V gene (*ATP6V0a2*) in mouse mammary glands leads to a reduction in glycosylation of ECM proteins that causes stiffness‐related alterations in breast tissue ECM. Upon tumor transplantation, these mice display an atypically soft, highly inflammatory and metastatic breast tumor phenotype. We also show the clinical significance of our results in patients with cancer where a2V expression levels in normal breast tissues inversely correlate with the metastasis incidence.

## 2 Materials and methods

### 2.1 Targeted disruption of the *Atp6v0a2* gene and mice

**Figure 1:**
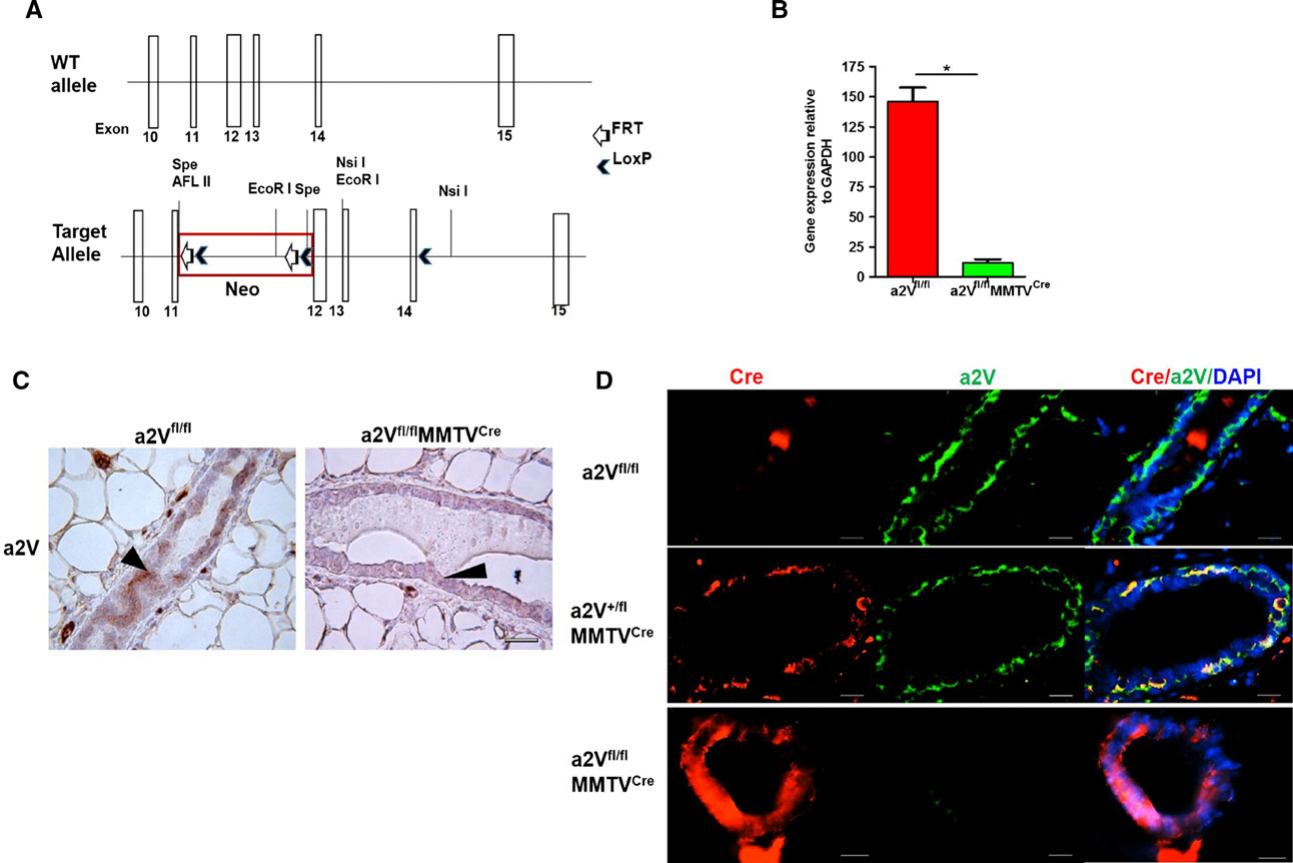
Mammary epithelial cell‐specific deletion of a2V gene. (A) Schematic of the wild‐type and floxed *ATP6V0a2* (a2V) gene. Exons 10–15 are shown with white boxes. The Lox/FRT‐Neo cassette was inserted upstream of exon 12 in an opposite direction relative to the a2V gene. A single LoxP site was inserted downstream of exon 14 in intron sequence. Some restriction enzyme sites are indicated. The presence of Cre and flox sites was confirmed by PCR (see Fig. S1A). (B) mRNA levels of a2 isoform in mammary epithelial cells isolated from breast tissues of a2V^fl/fl^ and a2V^fl/fl^ MMTV^C^ ^re^ mice. *n* = 15, *P <* *0.01. GAPDH was used as an endogenous control for normalization. The results are presented as mean ± SE. (C) Representative image showing a2V protein staining in mammary glands by immunohistochemistry (IHC) in breast tissues of a2V^fl/fl^ or a2V^fl/fl^ MMTV^C^ ^re^ mice using anti‐a2V antibody. Brown color shows positive staining for a2V in mammary epithelial cells (black arrow head), and blue color shows nuclear staining by counterstain hematoxylin. *n* = 8, magnification 40×, scale bar 50 μm. (D) Representative image showing staining of Cre recombinase (red) and a2V (green) proteins in mammary glands of wild‐type (a2V^fl/fl^), heterozygote (a2V^fl/+^ MMTV^C^ ^re^), and a2V‐knockout (a2V^fl/fl^ MMTV^C^ ^re^) mice by immunofluorescence analysis (IFA). *n* = 8, magnification 20×, scale bar 100 μm.

Floxed *ATP6v0a2* (a2V^fl/fl^) mice were generated as described before (Pamarthy *et al*., 2016). For conditional removal of *ATP6v0a2* gene, a2V^fl/fl^ mice were crossed with MMTV^Cre^ transgenic mice (Jackson Laboratories, Bar Harbor, ME, USA) resulting in a2V^fl/+^MMTV^Cre^ mice. The MMTV^Cre^ transgenic mice carry *Cre* recombinase under the control of regulatory promoter for the mouse mammary tumor virus (MMTV) long terminal repeat, which is specifically expressed in mammary epithelium. The presence of a2V^fl^ gene was confirmed by PCR by using the following primers: forward 5′‐AGGGTGGTGTCCTTTCACTCT and reverse 5′‐ATCCCCAGGATCCACGCAT (Fig. 1C). Further, a2V^fl/+^MMTV^Cre^ mice were backcrossed with a2V^fl/fl^ mice in order to obtain a2V^fl/fl^MMTV^Cre^ mice in which *ATP6v0a2* was specifically removed in mammary glands. Breast tissues from a2V^fl/fl^MMTV^Cre^ and a2V^fl/fl^ mice were used for *ATP6v0a2* RNA and protein analyses. All the animal experiments were performed in accordance with the Institutional Animal Care and Use Committee of the Rosalind Franklin University of Medicine and Science, North Chicago, Illinois.

### 2.2 Antibodies and reagents

Monoclonal anti‐a2V Ab was generated as described previously (Derks and Beaman, 2004). Rabbit polyclonal anti‐CK14 Ab, anti‐TNF‐α, anti‐collagen IV Ab, anti‐laminin1 + 2 Ab, anti‐GL25D2 Ab, Mu monoclonal anti‐EEA‐1 Ab and mouse anti‐β‐actin Ab were purchased from Abcam (Cambridge, MA, USA); rabbit anti‐GAPDH was from Cell Signaling (Danvers, MA, USA); and rabbit polyclonal anti‐giantin Ab from Biolegend (San Diego, CA, USA). Rabbit and mouse IgG isotype controls were purchased from Sigma (St Louis, MO, USA). EnVision+Dual Link System‐HRP polymer was obtained from DAKO (Glostrup Municipality, Denmark) and Alexa Fluor^®^ 488 and/or Alexa Fluor^®^ 594‐conjugated secondary antibody was purchased from Invitrogen. Permount from Fisher scientific and ProLong^®^ Gold containing DAPI (Invitrogen, Grand Island, NY, USA) were used as mounting media.

### 2.3 Cell lines and tissue samples

Mouse E0771 cells (CH3 Biosystems, Amherst, NY, USA) and Py230 mouse mammary carcinoma (ATCC, Manassas, VA, USA) were used for transplanting tumors in mice. E0771 was maintained as a monolayer in RPMI 1640 (Invitrogen) supplemented with 10% bovine serum, 100 U·mL^−1^ penicillin, 100 U·mL^−1^ streptomycin, and 10 mm HEPES buffer at 37 °C and 5% CO_2_. F12K medium (ATCC) was used for culturing Py230 cell line. Human mammary primary epithelial cells (HMEpC) (Cell Application, San Diego, CA. USA) were cultured in human mammary epithelial cell growth medium in the above‐mentioned conditions. Commercially available human breast tumor and matched normal breast tissue paraffin sections were procured from BioChain. GFP‐expressing E0771 cells were generated by transfecting with a phCMV‐GFP fusion stable vector (Genlantis, San Diego, CA, USA) and procedures according to the manufacturer's instructions.

### 2.4 Isolation of mammary epithelial cells

Mammary glands were dissected from female mice and were minced and dissociated enzymatically at 37 °C for 2 h in culture medium (DMEM/F12) containing 2% fetal bovine serum supplemented with 300 U·mL^−1^ collagenase and 100 U·mL^−1^ hyaluronidase (Stem Cell Technologies, Vancouver, Canada). The resulting cell suspension was suspended in 0.25% trypsin/EDTA for 2 min and centrifuged (250 ***g*** for 10 min). The resulting pellets were digested at 37 °C for 1 h in culture medium supplemented with dispase (2 mg·mL^−1^) and DNase (0.1 mg·mL^−1^) (Stem Cell Technologies). Dissociated cells were then depleted of red blood cells by suspending in RBC lysis buffer for 3 min and finally filtered through a 40‐mm mesh.

### 2.5 Histology, immunohistochemistry, and immunofluorescence

Tissue sections of 5 μm size from the fixed frozen breast tumors and paraffin‐embedded normal breasts were used. For histology, sections were stained with Mayer's hematoxylin and 0.1% eosin. Immunohistochemistry (IHC) was performed using Dako EnVision+ HRP‐DAB system in accordance with the manufacturer's instructions. Briefly, fixed frozen sections were boiled in sodium citrate buffer (pH = 6.0) for antigen retrieval. These sections were blocked for endogenous peroxidase activity by using dual peroxidase block and for protein blocking by using 5% BSA. Tissue sections were incubated with primary antibodies overnight at 4 °C followed by washing with PBST and incubation with secondary antibody polymer for 20 min at room temperature. DAB was used as a chromogen to detect specific proteins in tissue sections. The sections were counterstained with Mayer's hematoxylin and mounted in Permount mounting medium. Tissue sections were visualized and pictures were taken in light microscope Leica ICC 50W (Leica Biosystems, Wetzlar, Germany). For paraffin‐embedded normal breast tissues, sections were deparaffinized in xylene and processed similarly as frozen tissue sections. For immunofluorescence analysis (IFA), tissue sections were processed similarly as IHC except the peroxidase blocking, secondary antibodies, and mounting media. Alexa Fluor^®^ 488 and/or Alexa Fluor^®^ 594‐conjugated secondary antibodies and DAPI congaing mounting media were used for IFA. Tissue sections were visualized and imaged in Nikon Eclipse TE2000‐S fluorescence microscope (Nikon, Tokyo, Japan). Images were analyzed using Nikon nis‐element software. For IFA of cell lines, cells were washed thrice with PBS, fixed with 4% paraformaldehyde for 15 min, and permeabilized with 0.1% Triton X‐100 in PBS for 10 min at room temperature. Nonspecific binding was blocked by incubation with 3% fetal bovine serum in PBS for 1 h at room temperature and then incubated overnight with primary antibodies at 4 °C. On the next day, after washing three times with PBST, cells were incubated with Alexa Fluor^®^ 488 and/or Alexa Fluor^®^ 594‐conjugated secondary antibodies in 3% FBS in PBS for 45 min at room temperature. The cells were again rinsed with PBST and mounted with ProLong^®^ Gold mounting medium containing DAPI and allowed to polymerize at room temperature for 24 h. Cells were visualized and imaged in Nikon Eclipse TE2000‐S fluorescence microscope. Images were analyzed using Nikon NIS‐Element software.

### 2.6 Atomic force microscopy (AFM)

Experimental procedures for AFM were followed from recently published studies on AFM use in measuring tissue stiffness (Plodinec *et al*., 2012). Excised tumor or normal breast tissue sections were collected and stored in ice‐cold Ringer's buffer. AFM was performed same or next day of tissue collection. Tissues were immobilized and flatten on petri plate using all‐purpose Krazy glue. AFM force measurements were taken at room temperature using a BioScope Resolve AFM (Bruker, Billerica, MA, USA) integrated onto an Axio Observer.D1m (Carl Zeiss, Oberkochen, Germany) inverted optical microscope. Detailed methodology of AFM analysis is provided in the Supporting Information. AFM force measurements were taken at room temperature using a BioScope Resolve AFM (Bruker) integrated onto an Axio Observer.D1m (Carl Zeiss) inverted optical microscope. The system allowed precise lateral positioning of the silicon nitride AFM probe over the target position. To reduce stress on tissues during AFM mechanical measurement, smooth‐curved colloidal probe was used for elasticity measurements with a diameter of ~ 900‐nm SiO_2_ bead and ~ 30‐nm gold coating on the backside of the silicon nitride triangular cantilever (Novascan Technologies, Ames, IA, USA). The deflection sensitivity was calibrated by repeated contact mode indentation on a clean glass slide (VWR International, Radnor, PA, USA) in PBS and the spring constant of the compliant AFM cantilever was measured to be *k *= ~ 0.0635 N·m^−1^ by thermal noise method (Hutter and Bechhoefer, 1993). The tip radius was determined postmortem by scanning electron microscope (SEM Hitachi SU8030). The loading–unloading process was conducted at about 1.5 μm·s^−1^ and accomplished within roughly 1 s, during which the applied load, *F*, was measured as a function of the vertical actuation displacement of the piezoelectric cell, *y*, with 1024 data points collected. A maximum load of ~ 2 nN was applied at each data point, in order to keep the indentations within the elastic range.

The AFM cantilever with spring constant of *k*, connected to the piezoelectric cell, is used to deform the tissue, while the interaction force, *F*, is detected based on the deflection of the cantilever, δ_c_. The raw AFM data are the relationship between force, *F*, and piezoelectric cell displacement, *y*, where *F *= *k *× δ_c_. As the AFM cantilever is compliant, it is bending into the opposite direction, δ_c_, while the sample is indented by δ_s_. Thus, raw AFM force–displacement between (*F* vs. *y*) needs to be converted to the relation between applied force and cell deformation (*F* vs. δ_s_) by correcting cantilever deflection from the piezo displacement (Wang *et al*., 2012a,b, 2015). According to the spherical Hertzian contact mechanical model, the constitutive relation for a rigid spherical probe with radius of *R*_AFM_ pressing vertically on an elastic half continuum with elastic modulus, *E*, and Poisson's ratio, ʋ = 0.50, is used to compute the cell elastic modulus, given by

(1) $$E=\frac{3}{4}\left(\frac{1-\nu^{2}}{R_{\mathrm{AFM}}^{1/2}}\right)\frac{\partial F}{\partial (\delta_{s}^{3/2})}.$$

An in‐house‐developed MATLAB (MathWorks, Inc., Natick, MA, USA) code was used to obtain elasticity maps by analyzing all 1024 force curves in each force‐volume mapping, where the fitting goodness values *R* ^2^ exceeded 0.9 in all curve fittings. A line of zero force was defined from the average deflection of points on the force–displacement curve corresponding to the positions of the cantilever when it was far away from the surface.

### 2.7 Trichrome staining and hydroxyproline assay

Six‐micrometer‐thick tissue sections were prepared from tumorous and normal breast tissues. Total collagen was stained using trichrome staining kit (Sigma) as per the manufacturer's instructions. To quantitate collagen content, levels of hydroxyproline were measured in tissues using Hydroxyproline assay kit (Sigma). Briefly, 10 mg of tumorous or normal breast tissues was homogenized in 100 μL of water and hydrolyzed in 12 m concentrated HCl at 120 °C for 3 h. Twenty‐five microlitre of supernatant was transferred to 96‐well plate and dried at 60 °C in an oven. Hundred microlitre of chloramine T/oxidation buffer mixture was added to each sample and standard well and incubated at room temperature for 5 min. Further, 100 μL of the diluted DMAB reagent was added to well and incubated for 90 min at 60 °C. Absorbance was taken at 560 nm.

### 2.8 Western blot

Total protein lysates were prepared using NP‐40 lysis buffer containing protease inhibitor. Cell lysates were centrifuged for 30 min at 4 °C at 16 000 ***g*** to remove cellular debris. Protein concentrations were determined using Bradford assay. Immunoblotting was performed under native conditions for anti‐collagen IV antibody (Abcam) using 40 μg of proteins. After primary antibody incubation, membranes were incubated with IR secondary antibody (Licor) and signals were detected using Odyssey^®^ infrared imaging system (LI‐COR Biosciences, Lincoln, NE, USA).

### 2.9 Glycosylation staining

For glycosylation staining, tissue sections were processed for deparaffinization and antigen retrieval as described above in IHC methodology. DIG glycan differentiation kit (Roche Applied Science, Indianapolis, IN, USA) was used for the detection of O‐ and N‐linked glycosylation. Two different digoxygenin (DIG)‐labeled plant lectins *Sambucus nigra agglutinin* (SNA), identifies N‐linked glycosylation, and *peanut agglutinin* (PNA), identifies O‐linked glycosylation, were used. Briefly, tissue sections were blocked with a blocking buffer and washed in Tris buffer saline, 0.5% Tween‐20 (TBST), and buffer 1 (TBS, 1 mm MgCl_2_, 1 mm MnCl_2_, 1 mm CaCl_2_, pH 7.5). After washing, the sections were incubated overnight with DIG‐labeled SNA (10 μg·mL^−1^) and PNA (40 μg·mL^−1^) lectins at 4 °C. After washing with TBST, the sections were incubated with 2 μL·mL^−1^ anti‐DIG‐alkaline phosphatase for 1 h at room temperature. Tissue sections were incubated with 20 μL·mL^−1^ nitro‐blue‐tetrazolium chloride/5‐bromo‐4‐chloro‐3‐indolyl‐phosphate (NBT/BCIP) staining solution in buffer 2 (0.1 m Tris/HCl, 0.05 m MgCl_2_, 0.1 m NaCl, pH 9.5) for 3–5 min at room temperature. Upon development of dark brown/blue color, the reaction was stopped using ultra‐pure water. Western blot was also performed using same DIG glycan kit. Equal amount of protein from normal breast tissues was used for SDS/PAGE. After protein transfer, membranes were blocked and processed using the same reagents and methods as described above for tissue sections.

### 2.10 *In vivo* tumor generation

Tumors were generated as described before (Katara *et al*., 2016). Briefly, E0771 or Py230 mouse mammary carcinoma cells (5 × 10^5^ in 50 μL PBS) were inoculated subcutaneously into the left fourth abdominal mammary fat pad of 6‐ to 8‐week‐old female a2V^fl/fl^, a2V^+/fl^MMTV^Cre^_,_ and a2V^fl/fl^MMTV^Cre^ mice.

### 2.11 siRNA mediated knockdown of a2V

HMEpC (3 × 10^5^) cells were plated in 8‐chamber slides and transfected with three independent anti‐a2V siRNA (a) rGrCrCrUrUrArUrGrArCrArUrArGrCrCrArArArUrArArUTC, (b) rArCrArArGrGrUrUrArArGrArArGrArUrArUrGrUrGrArUTG, and (c) rArGrCrUrGrUrCrArArArUrCrArArGrArUrGrArUrGrGrGAA) and scrambled control siRNA (OriGene, Rockville, MD, USA). Lipofectamine 3000 RNAiMAX (Invitrogen) was used as a transfection reagent according to the manufacturer's instructions.

### 2.12 Statistical analysis

Statistical significance was calculated for each subset by Mann–Whitney test/unpaired *t*‐test using GraphPad PRISM 5 (GraphPad Software Inc., La Jolla, CA, USA). Collagen density was calculated as the proportion of blue collagen staining in each field of view in the Masson's trichrome staining using imagej (National Institutes of Health, Rockville, MD, USA). Sample size for animal experiments was determined by power calculation. Sample size for animal experiments was determined by power calculation. The investigators were not blinded to sample identity or results.

## 3 Results

### 3.1 Tumors in a2V‐knockout breast tissues exhibit soft phenotype and increased metastasis

To understand the role of host‐associated a2V in breast cancer, we generated mammary epithelial cell‐specific a2V‐knockout (a2V^fl/fl^MMTV^Cre^) mice (Fig. 1A, Fig. S1A). We observed a significant reduction in a2V mRNA levels in epithelial cells, isolated from mammary glands of a2V^fl/fl^MMTV^Cre^ mice compared to wild‐type (a2V^fl/fl^) mice (Fig. 1B). The deletion of a2V in a2V^fl/fl^MMTV^Cre^ mice did not alter gene expression of any other isoform of ‘a’ subunits (a1, a3, and a4) in the mammary gland (Fig. S1B). At the protein level, a2V was specifically deleted in the mammary epithelia of breast tissue as determined by immunohistochemistry (IHC) (Fig. 1C). In IFA, the absence of a2V protein (green) in a2V^fl/fl^MMTV^Cre^ mice confirms the deletion of floxed a2V gene (a2V^fl/fl^) by Cre recombinase (red) (Fig. 1D). These a2V^fl/fl^MMTV^Cre^ mice were followed up to 1 year of age to check for viability, fertility, and any sign of external abnormality due to the a2V deletion. The vital organs including heart, lungs, liver, spleen, and intestine appeared normal (data not shown).

**Figure 2:**
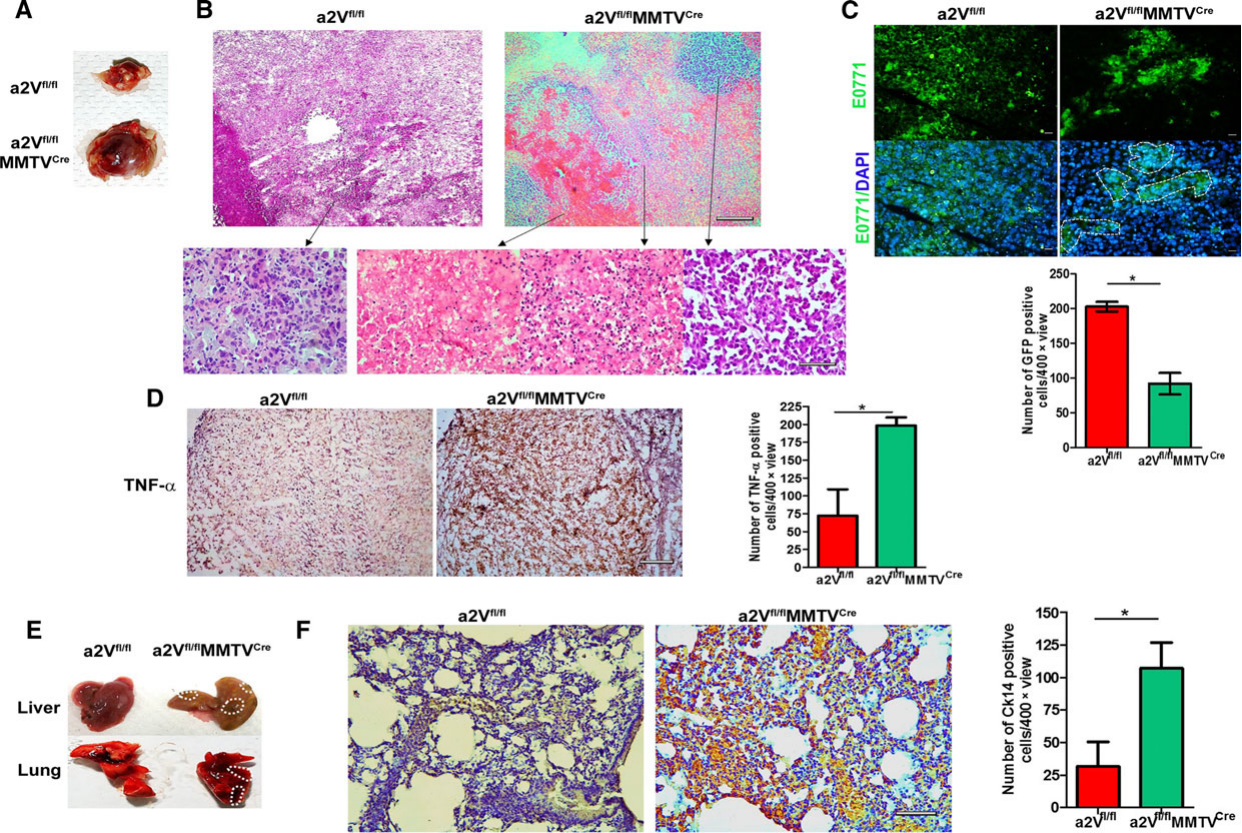
Tumor developed in a2V‐knockout mice shows inflammatory tumor microenvironment and increased metastases. (A) Representative images of breast tumors excised from a2V^fl/fl^ or a2V^fl/fl^ MMTV^C^ ^re^ mice at day 27. (B) Representative images of hematoxylin and eosin staining of tissue sections prepared from a2V^fl/fl^ or a2V^fl/fl^ MMTV^C^ ^re^ mice breast tumors. Upper panel shows overall histology at lower magnification (4×), scale bar 500 μm. Representative images shown in lower panel (left to right) indicate cancer cells in a2V^fl/fl^ tumors, and cancer cells, necrotic cells, and accumulation of RBCs in a2V^fl/fl^ MMTV^C^ ^re^ tumors at higher magnification (40×), scale bar 50 μm. (C) Representative GFP imaging of tissue section of breast tumors after E0771‐GFP cells were inoculated into a2V^fl/fl^ or a2V^fl/fl^ MMTV^C^ ^re^ mice. GFP‐positive cancer cells were visualized and quantified by fluorescent microscopy. In a2V^fl/fl^ MMTV^C^ ^re^ tumor sections, areas of restricted foci of cancer cells are highlighted by white dotted lines. Magnification 40×, scale bar 50 μm. Graph shows quantification of GFP‐positive cells in breast tumors from a2V^fl/fl^ or a2V^fl/fl^ MMTV^C^ ^re^ mice. Total number of GFP‐positive cells per 40× view were counted. Mean of total 10 views was considered per sample. Values are presented as mean ± SE,* n* = 7 each group. *P < 0.05. (D) Representative image showing TNF‐α staining (brown color) by IHC in tumor sections from a2V^fl/fl^ or a2V^fl/fl^ MMTV^C^ ^re^ mice, Magnification 10×, scale bar 200 μm. Graph shows quantification of TNF‐α‐positive cells. Positive cells per 400× view were counted. Mean of total 10 views was considered per sample. Values are presented as mean ± SE,* n *= 7 each group, *P < 0.01. (E) Representative images of lungs and livers harvested from tumor‐bearing a2V^fl/fl^ or a2V^fl/fl^ MMTV^C^ ^re^ mice showing metastatic lesions highlighted by dotted lines. (F) Ck14 staining by IHC in lung sections from tumor bearing a2V^fl/fl^ or a2V^fl/fl^ MMTV^C^ ^re^ mice, magnification 20×, scale bar 100 μm. Graph shows quantification of Ck14‐positive cells in breast tumors from a2V^fl/fl^ or a2V^fl/fl^ MMTV^C^ ^re^ mice. Total number of Ck14‐positive cells per 40× view were counted. Mean of total 10 views was considered per sample. Values are presented as mean ± SE,* n* = 7 each group.

To determine how the absence of a2V in mammary glands influences breast cancer growth, we used a transplant tumor model. E0771 and/or Py230 mouse mammary adenocarcinoma cells were inoculated into the mammary glands of a2V^fl/fl^MMTV^Cre^, a2V^fl/+^MMTV^Cre^, and a2V^fl/fl^ mice. E0771 cells generate tumors that are phenotypically and functionally similar to human breast tumors (Ewens *et al*., 2005). Py230 cells are derived from MMTV‐PyMT (mouse mammary tumor virus promoter‐driven polyoma middle T‐antigen) spontaneous tumor mouse and a well‐established model for studying metastasis (Biswas *et al*., 2014). In contrast to the solid primary breast tumor morphology typically observed in a2V^fl/fl^ mice, tumors in a2V^fl/fl^MMTV^Cre^ mice appeared softer, more vascularized, and hemorrhagic with serous fluid (Fig. 2A).

The phenotypic differences between these tumors were further confirmed by histopathological evaluations. The results revealed that tumors from a2V^fl/fl^ mice displayed a confluent sheet of viable tumor cells with microscopic foci of tumor necrosis. A dense infiltrate of neoplastic cells invading into adjacent adipose tissue and a scattered infiltrate of neutrophils and mononuclear cells were present (Fig. 2B). In contrast, a2V^fl/fl^MMTV^Cre^ tumors showed residual microscopic nests of viable tumor cells. Many sections revealed extravasated red blood cells with large zones of hemorrhagic necrosis. A dense inflammatory infiltrate including neutrophils and mononuclear cells was also observed, suggestive of restricted cancer cell proliferation in local microenvironment (Fig. 2B). To confirm this phenomenon, we examined cancer cells in tumor tissues generated by the inoculation of GFP‐tagged E0771 cells in mice. IFA of tissue sections confirmed a constricted area of focus with a significantly low number of GFP‐positive cancer cells at the primary tumor site in a2V^fl/fl^MMTV^Cre^ mice (Fig. 2C, *P* = 0.007). To confirm necrosis in tumors, we analyzed TNF‐α expression in tumor sections by IHC. Tumor tissues from a2V^fl/fl^MMTV^Cre^ mice showed significantly higher numbers of TNF‐α‐positive cells compared to control (Fig. 2D, *P* = 0.007), confirming an increased necrosis in tumor microenvironment in the absence of a2V. More interestingly, these mice showed increased metastasis of tumors compared to control as evident by liver and lung tissue lesions and cytokeratin (Ck)14 staining of lung tissue (Fig. 2E,F, Fig. S2). Quantitation of Ck14 staining in lung tissues showed 3.3‐fold increase in Ck14‐positive cells in a2V^fl/fl^MMTV^Cre^ mice compared to control (Fig. 2F, *P* = 0.004). These data indicated the localized inhibition of tumor cell proliferation in the absence of host a2V. However, these tumors were more invasive and metastatic, suggesting the involvement of host microenvironment in metastasis. Therefore, we aimed to characterize the composition of ECM in normal breast tissues of a2V^fl/fl^MMTV^Cre^ mice.

### 3.2 a2V deletion in mammary glands causes alterations in structure and stiffness of ECM

**Figure 3:**
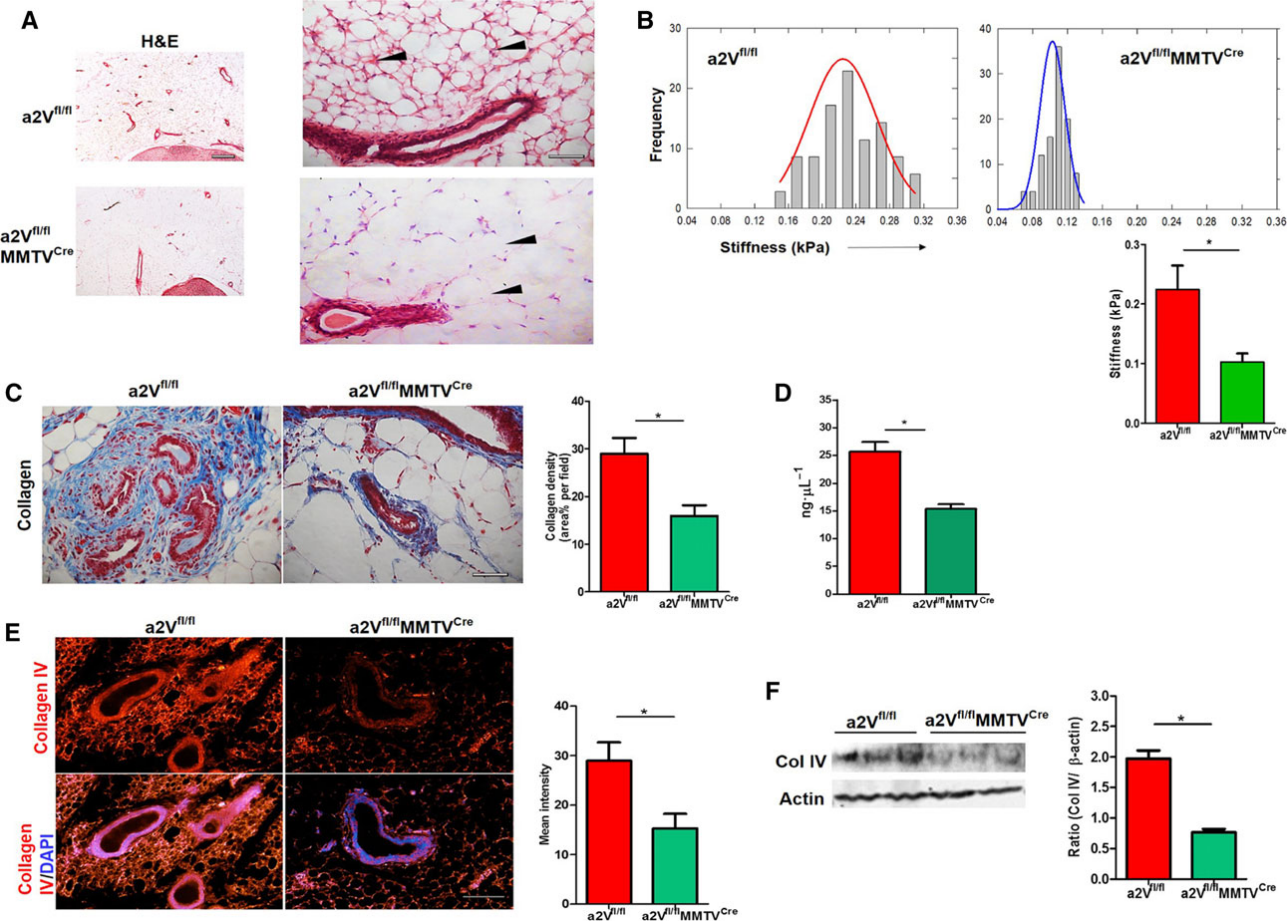
Breast tissue ECM of a2V‐knockout (a2V^fl/fl^ MMTV^C^ ^re^) mice displays reduced density and stiffness: (A) Representative images showing ECM density difference (black arrow head) in breast tissue sections from a2V^fl/fl^ or a2V^fl/fl^ MMTV^C^ ^re^ mice by hematoxylin and eosin staining. *n* = 8, magnification 4× and 20×, scale bar 500 and 100 μm. (B) Stiffness distribution of normal breast tissues from a2V^fl/fl^ or a2V^fl/fl^ MMTV^C^ ^re^ mice measured by atomic force microscopy. Single peaks demonstrate characteristic unimodal stiffness profile of normal breast tissue. Tissue stiffness data are represented in kPa, mean ± SD,* n* = 5, *P < 0.05. (C) Representative images of Mason trichrome staining of total collagen content in normal as well as tumorous breast tissues from a2V^fl/fl^ or a2V^fl/fl^ MMTV^C^ ^re^ mice. The blue color shows staining for collagen protein, and red color shows cytoplasm. *n* = 7 each group, magnification 10×, scale bar 200 μm. Graph shows quantification of collagen density. Values are presented as mean ± SE,* n* = 7 each group, *P <* *0.05. (D) Graph shows total hydroxyproline amino acid content in normal as well as tumorous breast tissues from a2V^fl/fl^ or a2V^fl/fl^ MMTV^C^ ^re^ mice measured by hydroxyproline assay. Values presented as mean ± SE,* n* = 12 and 15, *P < 0.05. (E) Representative images of immunofluorescence analysis of collagen IV protein expression in normal breast tissue sections from a2V^fl/fl^ or a2V^fl/fl^ MMTV^C^ ^re^ mice. The red color shows positive staining for collagen IV protein, and blue color shows DAPI staining for the nucleus, magnification 10×, scale bar 200 μm. (F) Western blot showing collagen IV protein expression in protein lysates prepared from a2V^fl/fl^ or a2V^fl/fl^ MMTV^C^ ^re^ mice breast tissues. *n *= 3 each group, *P < 0.05. Protein concentrations were normalized using β‐actin.

We next examined the normal breast tissues from a2V^fl/fl^MMTV^Cre^ mice for any structural changes due to a2V deletion. Histological evaluation revealed a clear difference in breast tissue architecture between the a2V^fl/fl^ and a2V^fl/fl^MMTV^Cre^ mice (Fig. 3A). In the a2V^fl/fl^ mice tissue, ductal structures were obvious, cell and ECM density was greater, while in a2V^fl/fl^MMTV^Cre^ mice there were a limited ductal development and thin ECM (Fig. 3A). These results suggested a thin and less dense ECM in a2V^fl/fl^MMTV^Cre^ mice. To further confirm the density difference in ECM, we analyzed the nanomechanical properties of breast tissues by atomic force microscopy (AFM). The results of AFM revealed a characteristic homogeneous unimodal stiffness distribution with a single peak in breast tissues from both a2V^fl/fl^MMTV^Cre^ and a2V^fl/fl^ mice. However, we observed lower stiffness values in a2V^fl/fl^MMTV^Cre^ breast tissues (0.10 ± 0.01 kPa) compared to t control (0.22 ± 0.04 kPa) (mean ± SD, *P *=* *0.001) (Fig. 3B). These data provide additional evidence that a2V deletion causes stiffness‐related alteration in a2V^fl/fl^MMTV^Cre^ mice.

Tumor stiffness is inversely correlated with cancer metastasis (Plodinec *et al*., 2012), and in a2V^fl/fl^MMTV^Cre^ mice, we observed more metastasis than a2V^fl/fl^ mice. Therefore, in addition to normal breast tissues, we also examined tumorous tissues for microscopic stiffness using AFM. Sequential stiffness maps across the specimen showed characteristic heterogeneity in tumor tissues from both a2V^fl/fl^MMTV^Cre^ and a2V^fl/fl^ mice. However, lesser stiffness as well as heterogeneity was observed in a2V^fl/fl^MMTV^Cre^ tumors as peak varies from 0.56 ± 0.11 kPa to 2.11 ± 0.16 kPa. In a2V^fl/fl^ tumors, peaks varied from 0.22 ± 0.18 kPa to 3.24 ± 0.22 kPa (mean ± SD) (Fig. S3). These data provide additional evidence that tumors in a2V knockout were softer and a2V deletion causes stiffness‐related alterations in breast tissues of these mice.

### 3.3 Loss of a2V reduces the production of ECM proteins by mammary epithelial cells

We next examined the status of ECM proteins as the possible reason for the compromised ECM production due to a2V deletion in mammary epithelial cells. In breast tissue ECM, collagen is the most abundant protein and collagen IV and laminins are important glycoproteins which are secreted by mammary epithelial cells to maintain the structural organization of ECM (Maller *et al*., 2010). Mason trichrome staining, which reacts with collagen, showed a significant decrease in the total collagen content in both normal and tumorous breast tissues of a2V^fl/fl^MMTV^Cre^ mice (Fig. 3C, *P* = 0.015 and Fig. S4A, *P *=* *0.03). In a2V^fl/fl^ mice, most of the collagen was organized into large tracks of aligned fibrils, whereas a2V^fl/fl^MMTV^Cre^ mice showed disorganized collagen bundles in breast tissues. Total collagen content was also measured by quantitating amount of hydroxyproline in tissue lysates prepared from breast tissues. Results of hydroxyproline assay confirmed a significant reduction in the collagen content in both normal (mean ± SE, 15.31 ± 0.8903 ng·μL^−1^, *n* = 15) and tumorous (31.15 ± 2.700, *n* = 5) breast tissues of a2V^fl/fl^MMTV^Cre^ mice compared to control (normal; 25.65 ± 1.773, *n* = 16, tumor; 57.15 ± 3.853, *n* = 5) (Fig. 3D, Fig. S4B). As a2V expression was inhibited in epithelial cells only, we examined the status of epithelial cell producing collagen IV and laminin proteins in these breast tissues. IFA of breast tissues revealed a significantly low production of collagen IV in a2V^fl/fl^MMTV^Cre^ mice (Fig. 3E, *P* = 0.01). Immunoblotting of collagen IV in protein lysates prepared from breast tissues also confirmed low production of collagen IV in a2V^fl/fl^MMTV^Cre^ mice (Fig. 3F, *P* = 0.03). Similarly, a low expression of laminin was also observed in breast tissues of a2V^fl/fl^MMTV^Cre^ mice (Fig. S4C, *P *=* *0.03). These results show that a2V deletion causes defects in ECM through reducing the production of matrix proteins required for proper composition.

### 3.4 Inhibition of a2V expression causes altered Golgi morphology and defective glycosylation in mammary epithelial cells

**Figure 4:**
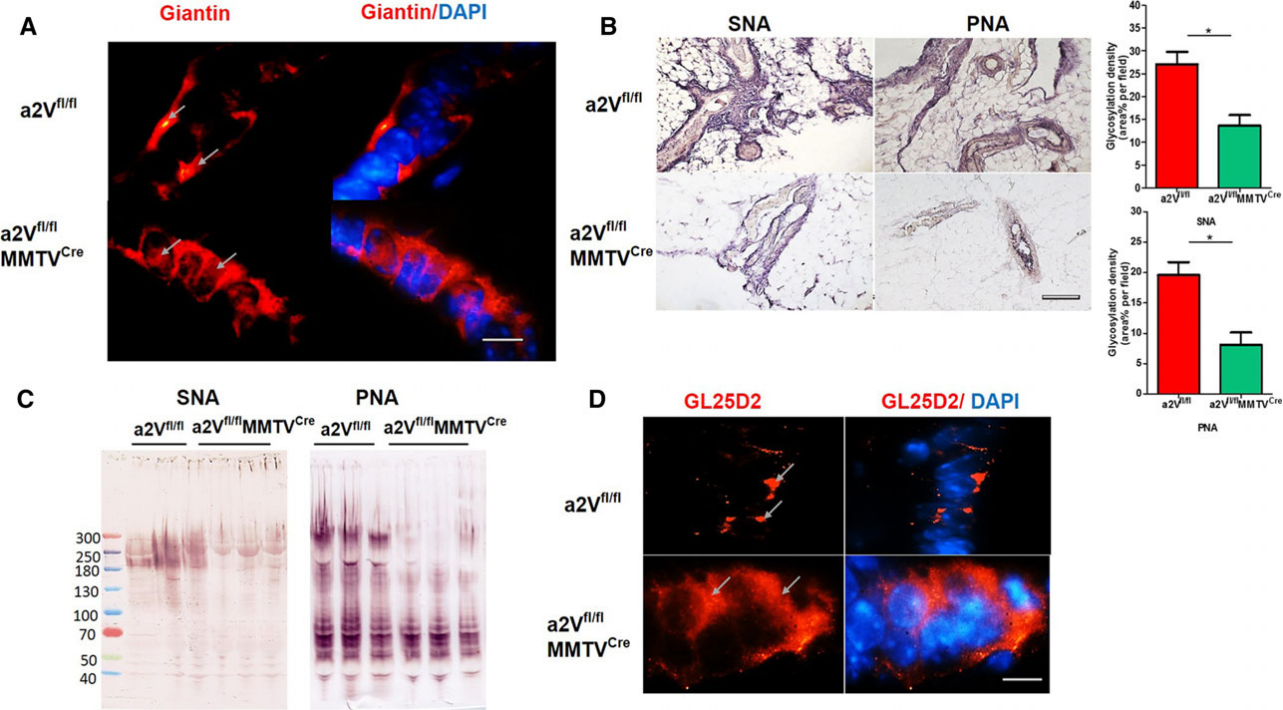
a2V deletion causes abnormal protein glycosylation and distribution of galactosyltransferase in Golgi: (A) IFA of Golgi morphology using anti‐giantin antibody in mammary glands of a2V^fl/fl^ or a2V^fl/fl^ MMTV^C^ ^re^ mice. Arrows indicate the perinuclear location of Golgi in mammary epithelial cells of a2V^fl/fl^ breast tissue, whereas in a2V^fl/fl^ MMTV^C^ ^re^ mice the Golgi exhibit dispersed morphology. *n* = 7 each group, magnification 100×, scale bar 20 μm. (B) SNA and PNA lectin staining demonstrating glycosylation in breast tissues from a2V^fl/fl^ or a2V^fl/fl^ MMTV^C^ ^re^ mice. The lectin staining appears as dark blue/brown color, magnification 10×, scale bar 200 μm. Graphs show glycosylation density per area field for SNA and PNA. Values are presented as mean ± SE,* n* = 7 each group, *P < 0.05. (C) Western blot analysis of glycosylation in protein lysates prepared from breast tissues of a2V^fl/fl^ or a2V^fl/fl^ MMTV^C^ ^re^ mice. *n* = 3 each group. (D) IFA of galactosyltransferase (GL25D2) location in mammary glands a2V^fl/fl^ or a2V^fl/fl^ MMTV^C^ ^re^ mice. Arrows show the perinuclear location of GL25D2 in mammary epithelial cells of a2V^fl/fl^ breast tissue, whereas in a2V^fl/fl^ MMTV^C^ ^re^ mice the enzyme exhibits distribution all over the cell. *n* = 5 each group, magnification 100×, scale bar 20 μm.

Next, we examined how the deletion of a2V leads to a low production of ECM proteins. a2V is present on Golgi membrane and disruption of a2V functions leads to deformation in Golgi structure (Axelsson *et al*., 2001; Udono *et al*., 2015). We evaluated Golgi morphology in mammary epithelial cells of breast tissues from a2V^fl/fl^MMTV^Cre^ mice by staining for giantin, which is a Golgi membrane protein. Results of IFA show a dispersed Golgi morphology in epithelial cells of mammary glands of a2V^fl/fl^MMTV^Cre^ mice compared to a typical perinuclear location in a2V^fl/fl^ mice (Fig. 4A). No generic increase in giantin protein expression was observed in a2V^fl/fl^MMTV^Cre^ mice due to a2V deletion (Fig. S5A). The structural deformation in Golgi is known to cause glycosylation defects in mammalian cells. Also, in ACRL patients, abnormal glycosylation of serum proteins is associated with loss‐of‐function mutation in the a2V gene (Fischer *et al*., 2012; Guillard *et al*., 2009; Kornak *et al*., 2008). To determine whether a similar phenomenon occurs in our model, we examined the protein glycosylation defects in ECM of normal breast tissue. During post‐translational modifications, proteins are glycosylated inside Golgi and carbohydrate chains are attached at specific positions to these proteins in the presence of glycosyltransferases (Breton *et al*., 2006). O‐ and N‐linked are the two major types of glycosylations and various mammary gland ECM proteins are glycosylated at these positions (Burchell *et al*., 2001; Vijay, 1998). Lectins SNA and PNA were used to identify O‐ and N‐linked protein glycosylation, respectively, in breast tissues. Results of glycoprotein IHC in breast tissues showed a significantly reduced staining for both SNA and PNA lectins in a2V^fl/fl^MMTV^Cre^ mice compared to control (Fig. 4B, *P* = 0.01, Fig. S5B). The glycosylation defects were also evaluated in mammary epithelial cells isolated from mice by immunoblotting. A reduction in glycosylation of proteins was also observed in protein lysates prepared from these cells by western blot (Fig. 4C).

Neutralization of Golgi pH causes relocation of glycosyltransferase enzymes from Golgi to endosomes or plasma membrane causing protein glycosylation‐related changes in cells (Axelsson *et al*., 2001; Udono *et al*., 2015). Therefore, we evaluated any change in the distribution of GL25D2, a galactosyltransferase that glycosylates collagen IV in Golgi, as a possible factor for glycosylation irregularities in a2V^fl/fl^MMTV^Cre^ mice. Results showed a dispersed distribution of GL25D2 throughout the cytoplasm in epithelial cells of a2V^fl/fl^MMTV^Cre^ mice breast tissues compared to control where it showed a perinuclear location (Fig. 4D). This finding suggests the reduced availability of GL25D2 in Golgi for efficient glycosylation of proteins processed therein. These data confirmed that glycosylation defects in ECM proteins in a2V^fl/fl^MMTV^Cre^ mice breast tissues are due to a2V deletion.

**Figure 5:**
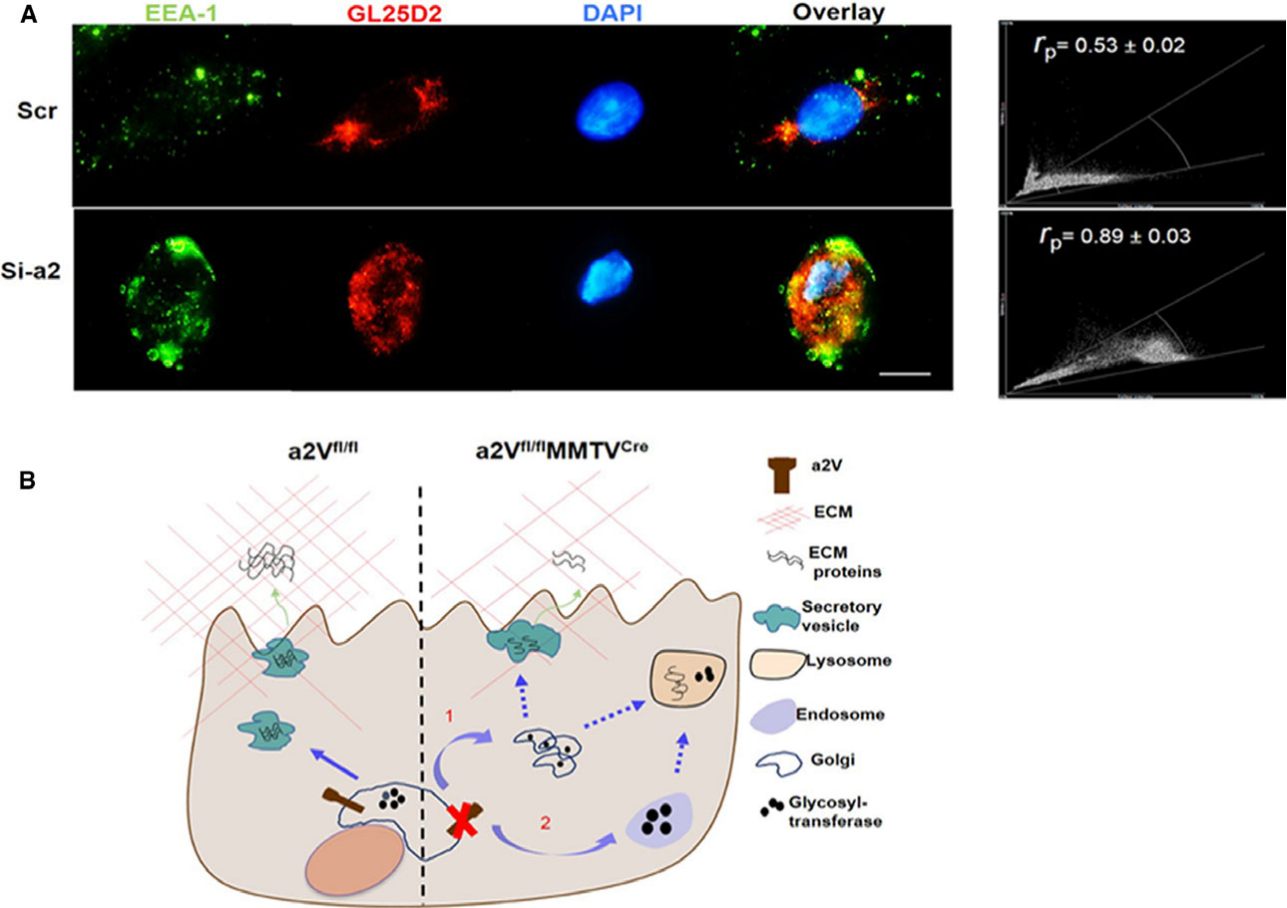
The loss of a2V causes relocation of galactosyltransferase from Golgi to endosome in mammary epithelial cells. (A) Representative images of colocalization of GL25D2 and EEA‐1 (early endosome marker) by IFA in HMEpC cells. To inhibit a2V expression, HMEpC cells were treated with scrambled RNA control (Scr) or three different anti‐a2V siRNAs (Si‐a2). Cells were then stained with anti‐EEA‐1 (green) and anti‐GL25D2 (red), magnification 100×, scale bar 20 μm. Efficiencies of all siRNAs were quantified by real‐time PCR (see Fig. S5). Pearson's correlation coefficient and scatter plot between EEA‐1 and GL25D2 are also shown in this panel. *n* = 3 for each siRNA. (B) Schematic representing regulation of ECM composition by V‐ATPase: under normal conditions (a2V^fl/fl^), Golgi pH is maintained through the function of a2V. ECM proteins are produced and properly glycosylated by glycosyltransferases; these proteins are transported to extracellular space through secretory vesicles where they contribute to ECM composition. When a2V function is interrupted (a2V^fl/fl^ MMTV^C^ ^re^), pH imbalance in Golgi results in a dispersed Golgi morphology (1) and relocation of glycosyltransferases to early endosomes and then to lysosomes (2). Due to the lack of effective glycosyltransferase activity in Golgi, ECM proteins are improperly glycosylated, which affects their production. These abnormally glycosylated proteins and glycosyltransferases are later transported to lysosomes for degradation.

We next examined the cellular location of GL25D2 after inhibition of a2V expression. We used human primary mammary epithelial cells (HMEpC) and inhibited a2V expression using three independent anti‐a2V siRNA. The efficiency of a2V knockdown was evaluated by quantitative RT‐PCR and western blot (Fig. S6). These transfected HMEpC cells were then stained for GL25D2, EEA‐1 (early endosome antigen), to determine GL25D2 distribution after a2V inhibition. We observed that a2V knockdown in primary mammary epithelial cells resulted in the relocation of GL25D2 to the early endosomes from perinuclear location (Fig. 5A, Pearson's correlation coefficient *r*_p _= 0.89 ± 0.03, *P *=* *0.01), confirming alteration in Golgi functioning. These results confirm that the inhibition of a2V expression in mammary epithelial cells causes abnormal Golgi morphology and relocation of glycosyltransferases to early endosome from Golgi. This results in defects in protein glycosylation and low production of ECM proteins that lead to the inadequate ECM formation (Fig. 5B).

### 3.5 Low a2V expression in normal breast is associated with metastatic disease in patients with breast cancer

**Figure 6:**
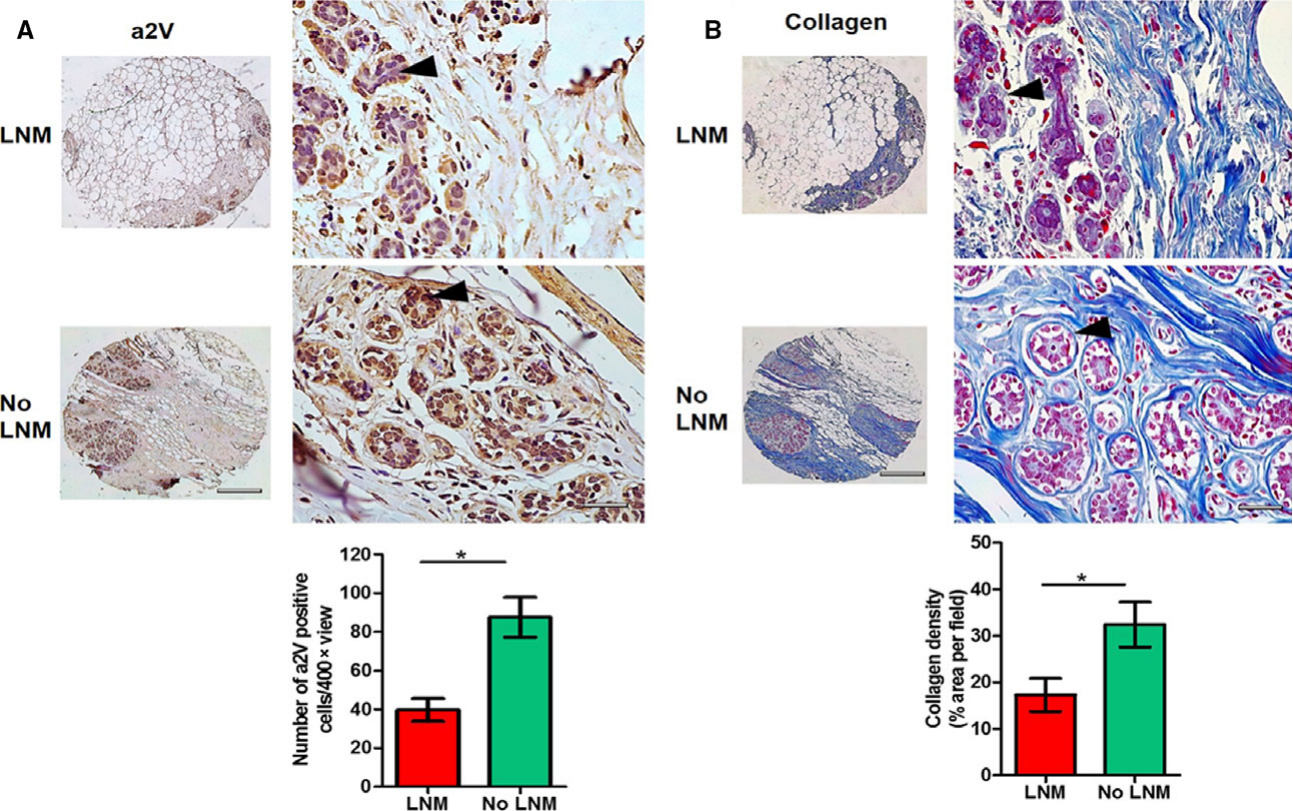
Evaluation of a2V expression and collagen protein in human normal breast: (A) representative IHC images showing a2V protein staining in normal or uninvolved breast tissues from breast cancer patients reported with lymph node metastasis (LNM) or no LNM of tumors. Brown color (black arrow head) shows positive staining for a2V, and blue color shows nuclear staining by counterstain hematoxylin. *n* = 5, left panel shows overall histology at lower magnification (4×), scale bar 500 μm. Representative images at higher magnification (40×) are shown in the right panel, scale bar 50 μm. Graph shows quantification of a2V‐positive cells in breast tumors from LNM and no LNM breast cancer patients. Total number of a2V‐positive cells per 40× view were counted. Mean of total 10 views was considered per sample. Values are presented as mean ± SE,* n* = 5 each group, *P < 0.05. (B) Representative images of Mason trichrome staining of total collagen content in same tissues. Black arrow head shows collagen sheets around mammary epithelial cells. Left panel shows overall histology at lower magnification (4×), scale bar 500 μm. Representative images at higher magnification (40×) are shown in the right panel, scale bar 50 μm. Graph shows quantification of collagen density. Values are presented as mean ± SE,* n *= 5 each group, *P <* *0.05.

To test the clinical relevance of our findings, we examined a2V expression levels in normal or uninvolved breast tissues from patients with cancer reported with lymph node metastasis (LNM) or no LNM of tumors. We observed that patients with LNM had a significantly lower expression of a2V in mammary glands of uninvolved or normal breast tissues compared to patients with no LNM of tumors (Fig. 6A, *P* = 0.02). The total collagen was also stained in these tissues to determine whether low a2V expression levels have any effect on collagen content or organization. Results of Mason trichrome staining showed a lower deposition of total collagen in ECM of normal breast tissues in patients with LNM compared to no LNM patients (Fig. 6B, *P* = 0.03). An inconsistency in collagen sheets around mammary epithelial cells was also observed in normal breast tissues from patients with LNM, suggesting stiffness‐related alterations in ECM (Fig. 6B). H&E, a2V, and collagen staining procedures of normal breast and respective primary tumor tissues from same patients are shown in Fig. S7. These results indicate that a2V expression in mammary glands has an impact on ECM architecture and evaluation of a2V levels in normal breast tissues can help in predicting the metastatic fate of tumor cells.

## 4 Discussion

Although V‐ATPase is essentially required for cancer progression and metastasis, relatively few studies have examined its functional role in breast cancer. The present study aimed to decipher the role of host‐associated V‐ATPase in breast cancer pathology. For the first time, this study provides an insight into the association of V‐ATPase with ECM architecture *in vivo* by employing a unique experimental mouse model. Here, we have established that the absence of a2V in mammary gland causes abnormality in ECM composition and architecture that further augment breast cancer dissemination.

A stiff or dense ECM stimulates the transformation of epithelial cells from normal to malignant cancer cells (Calvo *et al*., 2013; Levental *et al*., 2009). It has been suggested that stiffer breast tissue tends to have a higher risk of breast cancer (McCormack and dos Santos Silva, 2006). The collagen protein content has been considered as a major contributor in ECM stiffness. However, in the mouse model, increased collagen cross‐linking, but not collagen content, enhances ECM stiffness and cancer malignancy (Levental *et al*., 2009). Therefore, the correlation between collagen content and cancer outcome and/or metastases is still not clear. Studies have shown either an inverse relationship or failed to establish any correlation in humans (Eriksson *et al*., 2013; Gierach *et al*., 2012; Kakkad *et al*., 2012; Wasuthit *et al*., 2011). In the mouse, linearized collagen bundles increase cell migration, whereas a dense network of stiff matrix fibers hampers migration (Egeblad *et al*., 2010). Here, in a2V^fl/fl^MMTV^Cre^ mice, we observed an altered and less stiff ECM of normal as well as tumorous breast tissue. We also observed less total collagen content and a lower expression of collagen IV and laminin in breast tissues of a2V^fl/fl^MMTV^Cre^ mice. In breast tissue ECM, stromal collagen I, a fibrillar collagen, is secreted by fibroblasts, whereas basement membrane collagen IV is secreted by mammary epithelial cells. The adequate structure and functioning of collagen is dependent on the proper production and anchoring of these molecules. Abnormal glycosylation or other changes in any protein can lead to dysfunction of collagen protein, which finally affects the adequate cross‐linking to other proteins and appropriate ECM architecture (Maller *et al*., 2010). Here, Masson's trichrome staining, which reacts with all fibrillar collagen, confirmed a significant reduction in fibril‐associated collagen in a2V^fl/fl^MMTV^Cre^ mice. We speculate that less secretion of collagen IV by epithelial cells can lead to the degradation of fibroblast‐secreted collagen I and results in the reduction in total collagen content in breast tissue. In basal lamina, collagen IV cross‐links with laminin through nidogen glycoproteins and any change in collagen production can affect its binding with laminin (Mouw *et al*., 2014). Further, like collagen IV, laminin is a glycosylated protein and secreted by epithelial cells. Here, the reduction in laminin expression can be a combinatorial effect of abnormal glycosylation in epithelial cells and the unavailability of collagen IV for cross‐linking in ECM. This decrease in laminin expression can also contribute to increased metastasis in a2V^fl/fl^MMTV^Cre^ mice as downregulation in laminin expression has been shown in various advanced breast cancers (Pouliot and Kusuma, 2013). In cutis laxa patients, mutation in V‐ATPase‐a2 gene leads to glycosylation defects in tropoelastin protein, which affects its secretion from fibroblast cells in skin. This tropoelastin protein further fails to create an adequate cross‐linking with collagen and fibronectin which are required for proper ECM structure in skin (Guillard *et al*., 2009; Hucthagowder *et al*., 2009). Loss of O‐glycosylation in epithelial cells reduces the secretion of basement membrane collagen protein that affects ECM composition in submandibular gland in mice (Tian *et al*., 2012). Further, upon inhibition of V‐ATPase function, Golgi pH and structure are disrupted and glycosyltransferases are relocated from Golgi to early endosomes or plasma membranes, resulting in glycosylation changes (Hucthagowder *et al*., 2009; Kellokumpu *et al*., 2002; Udono *et al*., 2015). Our results are consistent with these studies as we found a similar glycosylation and compositional defects in ECM and dispersed Golgi morphology in mammary epithelial cells of a2V^fl/fl^MMTV^Cre^ mice breast tissues. In addition, siRNA inhibition of a2V in epithelial cells results in relocation of GL25D2 to early endosomes, confirming the a2V‐mediated protein glycosylation defects in a2V^fl/fl^MMTV^Cre^ mice.

Cancer cells adhere to the ECM through various receptors and an abnormal ECM composition can lead to the failure in the establishment of a solid primary tumor phenotype (Lu *et al*., 2012). Here, a2V^fl/fl^MMTV^Cre^ mice produced soft primary tumors and restricted cell proliferation as evident by the decreased number of GFP‐positive cancer cells. Additionally, the presence of high TNF‐α expression and necrotic tissue suggests greater‐than‐normal inflammatory responses in the local microenvironment in a2V^fl/fl^MMTV^Cre^ mice. In a murine model of pancreatic cancer, SPARC gene deficiency caused a reduction in expression levels of types I, III, and IV collagen that led to elevated tumor metastases (Arnold *et al*., 2010). We speculate that due to the inadequacy of collagen and other proteins in ECM of a2V^fl/fl^MMTV^Cre^ breast tissues, cancer cells failed to establish as solid primary tumors and inflammation at tumor sites restricted their localized growth. The altered ECM in these mice further helps cancer cells to escape inflammation and migrate to distant organs. The higher inflammatory infiltrate of leukocytes accompanied by increased metastases in a2V^fl/fl^MMTV^Cre^ mice suggests an easy infiltration of immune cells as well as the migration of cancer cells from the tumor implantation site. In a2V^fl/fl^MMTV^Cre^ mice, the soft tumor phenotype, low collagen content, and increased metastasis correlate with recent murine breast cancer studies showing that a ‘soft’ tumor tissue phenotype and low collagen content correspond to increased metastases (Fenner *et al*., 2014; Plodinec *et al*., 2012).

Clinically, we found that a2V expression may serve as a novel biomarker for breast cancer. a2V is highly expressed in primary tumors of patients with breast cancer (Fig. S6). Although there was a limited sample size for paired tissues from patients with breast cancer, we demonstrated that lower expression of a2V in normal or uninvolved breasts tissues correlates with the collagen disorganization in patients with metastatic breast cancer. In normal conditions, expression of various isoforms of V‐ATPase was regulated at both transcriptional and post‐transcriptional levels. Basal expression of V‐ATPase ‘a3’ isoform has been shown to be suppressed by poly(ADP‐ribose) polymerase‐1 (PARP‐1), a highly conserved nuclear protein that modulates chromatin structure and causes transcriptional inhibition (Beranger *et al*., 2006; Serrano *et al*., 2009). The involvement of 3′ untranslated regions and AU‐rich element has been shown in post‐transcriptional regulation of various V‐ATPase isoforms (Jeyaraj *et al*., 2005; Wang *et al*., 2002). A genome‐wide analysis study revealed higher‐efficiency miRNA binding sites in mRNA of ‘a1’ isoform of V‐ATPase (O'Connor *et al*., 2008). This miRNA predicted to cause a reduction in a1 translation that further impairs the regulation of secretory pathway. The reduction in a2V mRNA levels in normal breast tissues of patients with LNM can be due to these factors that need to be investigated. The clinical significance of this finding warrants further investigation to determine particularly whether a2V expression defines a group of patients with risk of developing metastatic disease and whether they thus may require personalized therapy.

## 5 Conclusions

For the first time, this study establishes the direct link between breast tissue ECM stiffness and V‐ATPase activity. These data show that host cell‐associated V‐ATPase expression levels can affect breast cancer dissemination, at least in part, through ECM remodeling. Considering the diverse role of ECM stiffness in cancer outcome, this study highlights a2V as an important regulatory molecule in cancer mechanics. The inclusion of a2V with other clinically relevant prognostic markers could efficiently help in the risk assessment of metastases and the designing of personalized therapy for patients with breast cancer.

## Author contributions

GKK and AK contributed to designing the research, performing the experiments, interpreting the data, making figures, and writing the manuscript. LM, MS, SI, and SP assisted in animal breeding and experiments for the study. XW and GSS assisted with AFM experiments and data analysis. KS analyzed and interpreted tissue pathology. AGS and KDB wrote and edited the manuscript and provided resources for study.

## Supporting information


**Fig. S1.** Genotyping PCR and mRNA expression analysis of other isoforms of ‘a’ subunit.
**Fig. S2.** Tumors in a2V^fl/fl^MMTV^Cre^ mice displayed increased metastasis.
**Fig. S3.** Stiffness profile of tumors.
**Fig. S4.** Collagen profile of tumors and expression analysis of laminin in normal breast tissue.
**Fig. S5.** (A) Western blot showing Giantin protein (~367kd) expression in protein lysates prepared from purified mammary epithelial cells from breast tissues of a2V^fl/fl^ or a2V^fl/fl^MMTV^Cre^ mice. Protein concentrations were normalized using β‐actin (45kd). (B) SNA lectin staining demonstrating glycosylation in breast tissues from a2V^fl/fl^ or a2V^fl/fl^MMTV^Cre^ mice. The lectin staining appears as dark blue/brown color, magnification 10×, scale bar 200 μm.
**Fig. S6.** siRNA mediated knockdown of a2V.
**Fig. S7.** Evaluation of a2V expression and collagen protein in human breast tissue: representative images of H&E, a2V and Mason Trichrome staining in paired normal breast and primary tumor tissue from breast cancer patients reported with lymph node metastasis (LNM) of no LNM of tumors (*n* = 5).
