# Supplementary material for: Mammary epithelium‐specific inactivation of V‐ATPase reduces stiffness of extracellular matrix and enhances metastasis of breast cancer
Source: Mol Oncol. 2017 Dec 12;12(2):208–23. doi: 10.1002/1878-0261.12159 (PMC5792725; doi:10.1002/1878-0261.12159)
Supplement: Supplementary file 1 — Fig. S1. Genotyping PCR and mRNA expression analysis of other isoforms of ‘a’ subunit. Fig. S2. Tumors in a2Vfl/flMMTVCre mice displayed increased metastasis. Fig. S3. Stiffness profile of tumors. Fig. S4. Collagen profile of tumors and expression analysis of laminin in normal breast tissue. Fig. S5. (A) Western blot showing Giantin protein (~367kd) expression in protein lysates prepared from purified mammary epithelial cells from breast tissues of a2Vfl/fl or a2Vfl/flMMTVCre mice. Protein concentrations were normalized using β‐actin (45kd). (B) SNA lectin staining demonstrating glycosylation in breast tissues from a2Vfl/fl or a2Vfl/flMMTVCre mice. The lectin staining appears as dark blue/brown color, magnification 10×, scale bar 200 μm. Fig. S6. siRNA mediated knockdown of a2V. Fig. S7. Evaluation of a2V expression and collagen protein in human breast tissue: representative images of H&E, a2V and Mason Trichrome staining in paired normal breast and primary tumor tissue from breast cancer patients reported with lymph node metastasis (LNM) of no LNM of tumors (n = 5). [file MOL2-12-208-s001.docx]

**Supplementary Information**

**Mammary Epithelium-specific Inactivation of V-ATPase Reduces Stiffness of Extracellular Matrix and Enhances Metastasis of Breast Cancer**

Gajendra Kumar Katara, Arpita Kulshrestha, Liqun Mao, Xin Wang, Manoranjan Sahoo, Safaa Ibrahim, Sahithi Pamarthy, Kimiko Suzue, Gajendra Singh Shekhawat, Alice Gilman-Sachs & Kenneth D. Beaman

**Fig. S1**


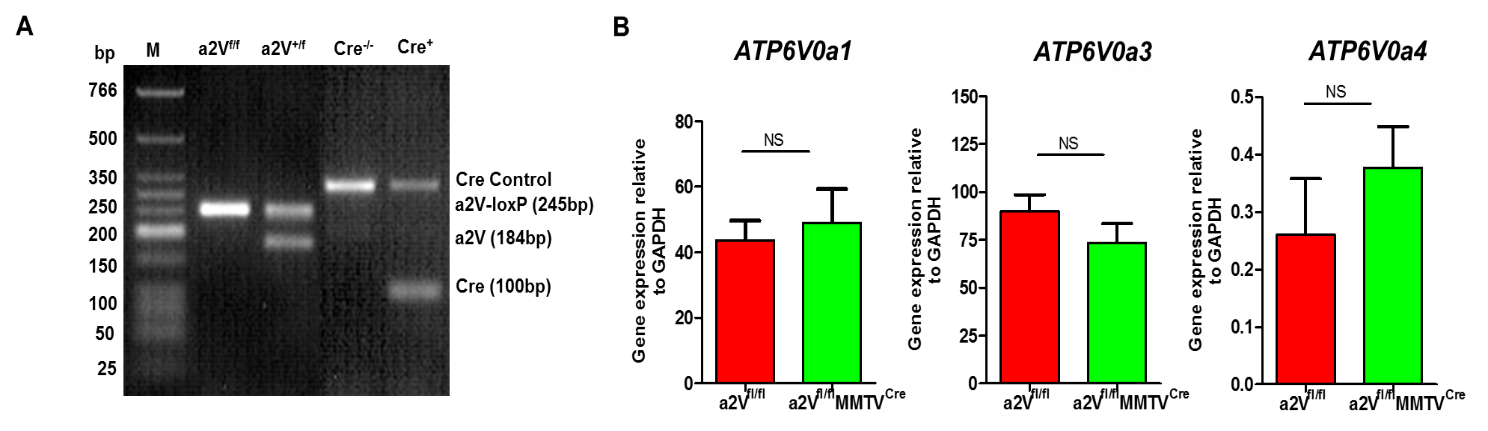


**Fig. S2**

**
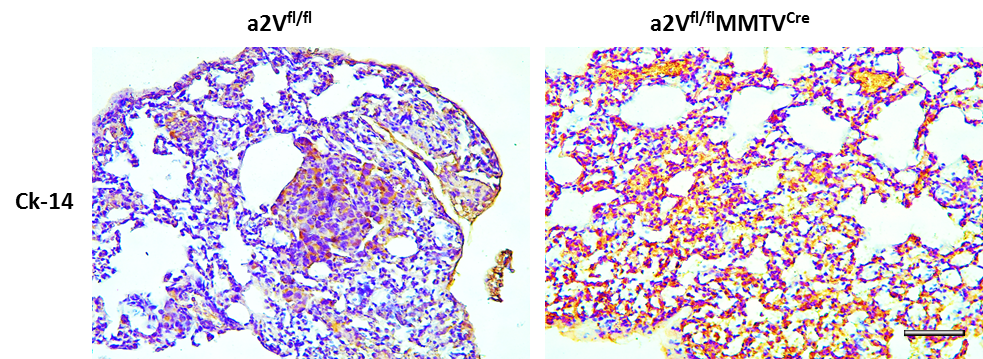
**

**Fig. S3**

**
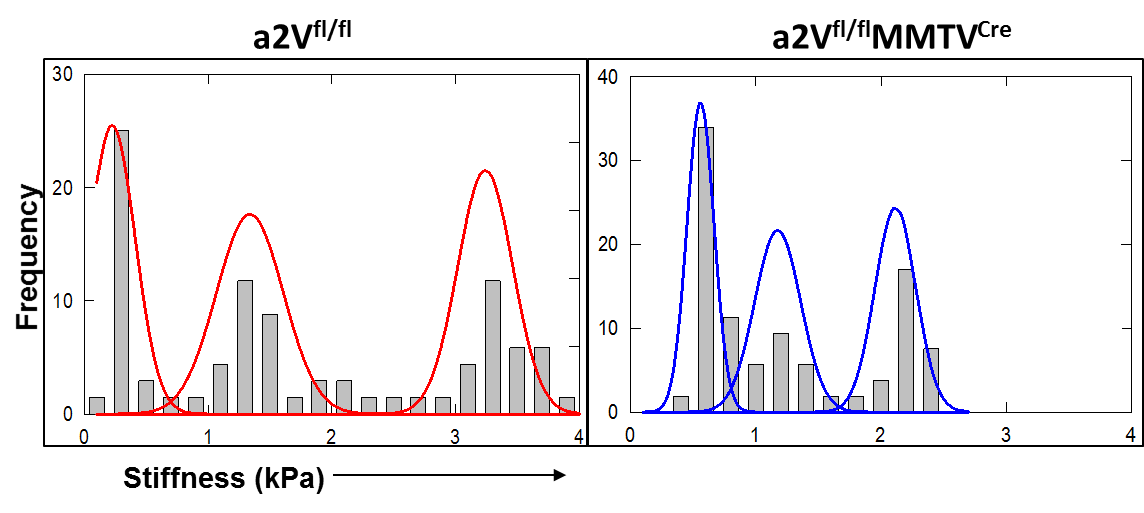
**

**Fig. S4**

**
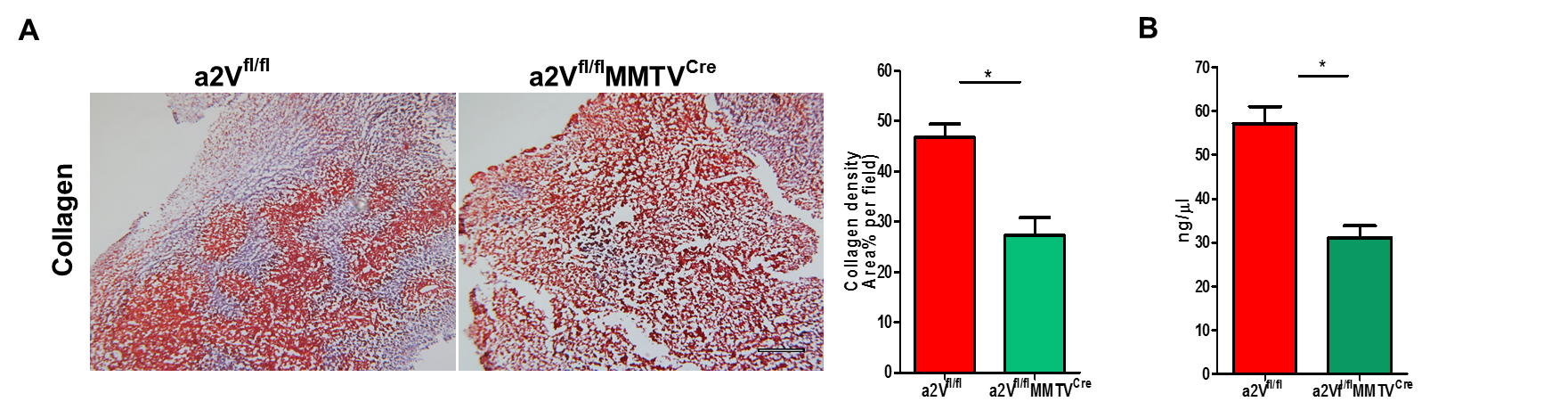
**

**
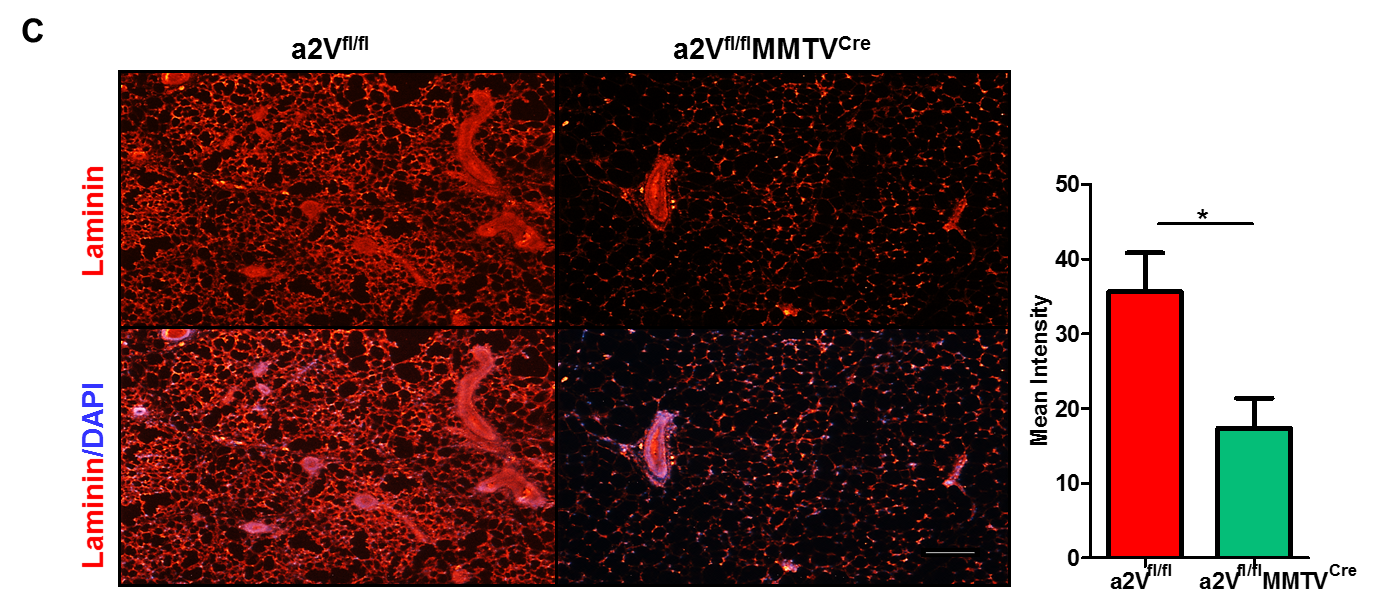
**

**Fig. S5**

**
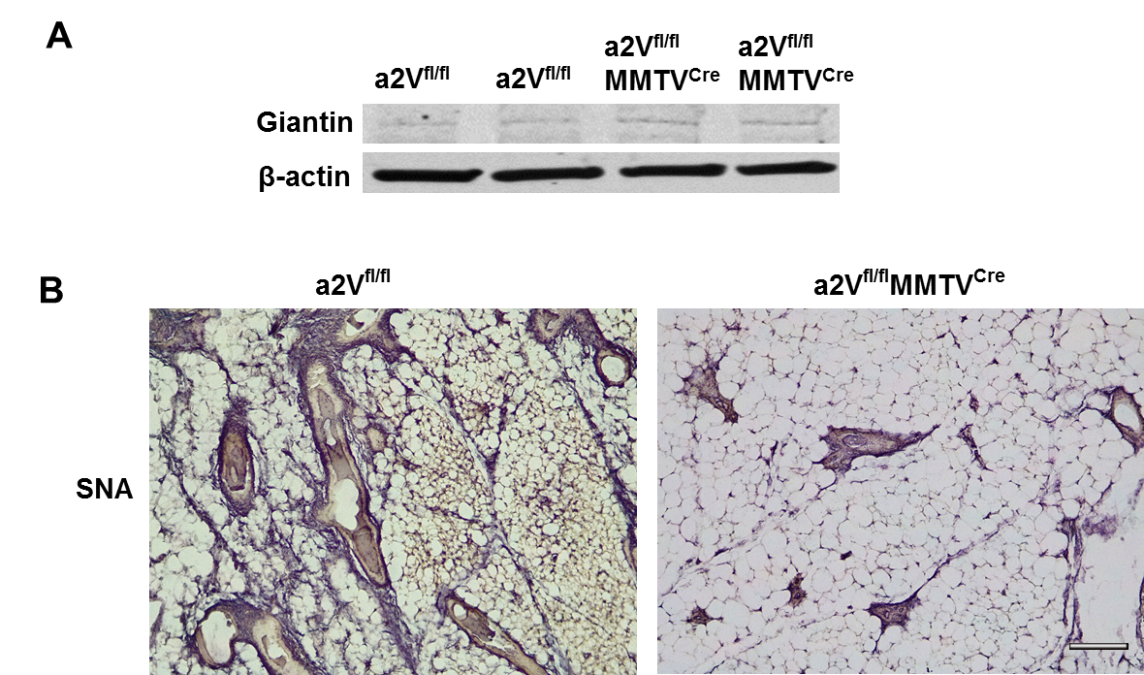
**

**Fig. S6**

**
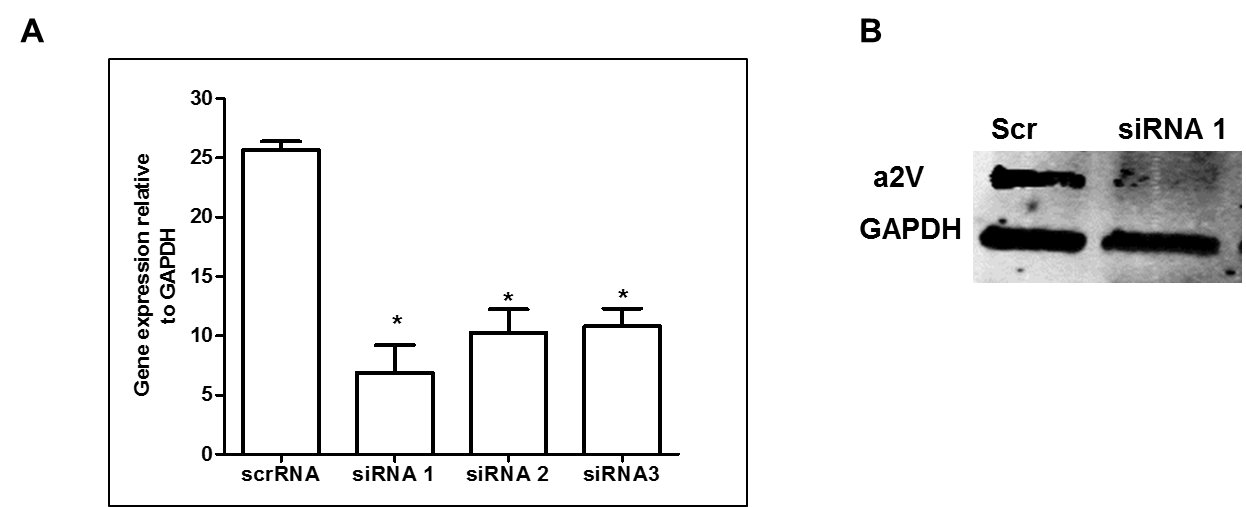
**

**Fig. S7**

**
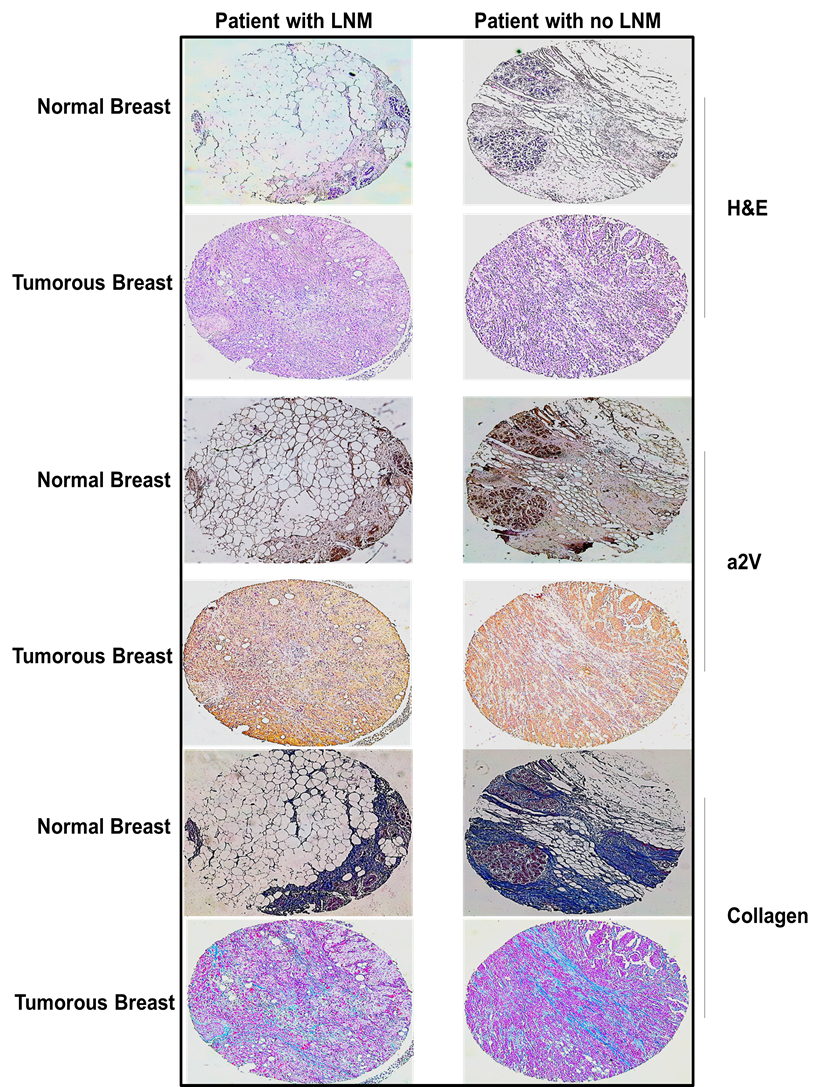
**
