# Supplementary material for: Mammary epithelium‐specific inactivation of V‐ATPase reduces stiffness of extracellular matrix and enhances metastasis of breast cancer
Source: Mol Oncol. 2017 Dec 12;12(2):208–23. doi: 10.1002/1878-0261.12159 (PMC5792725; doi:10.1002/1878-0261.12159)
Supplement: Supplementary file 2 [file MOL2-12-208-s002.docx]

**Figure legends for supplementary figures**

**Fig. S1: Genotyping PCR and mRNA expression analysis of other isoforms of ‘a’ subunit. (A)** The presence of Cre and Flox sites was confirmed by PCR in genomic DNA. PCR showing floxed a2V (245bp), a2V (184bp) and Cre (100bp) products. **(B)** mRNA levels of a1, a3 and a4 isoforms of ‘a’ subunit of V-ATPase in mammary epithelial cells isolated from breast tissues of a2V^fl/fl^ and a2V^fl/fl^MMTV^Cre^ mice. n=15, NS = not significant. GAPDH was used as an endogenous control for normalization. The results are presented as mean ± SE.

**Fig. S2: Tumors in a2V^fl/fl^MMTV^Cre^ mice displayed increased metastasis**. Py230 mouse mammary carcinoma cells were inoculated into the abdominal mammary fat pad of female a2V^fl/fl^ or a2V^+/fl^ or a2V^fl/fl^MMTV^Cre^ mice (n =8 each group). Representative image of Ck14 staining by IHC in lung sections from tumor bearing a2V^fl/fl^ and a2V^fl/fl^MMTV^Cre^ mice. Brown color shows proliferating cancer cells in lung tissue. Magnification 4X, scale bar 500µm.

**Fig. S3:** **Stiffness profile of tumors**. Stiffness distribution of tumorous breast tissues from a2V^fl/fl^ or a2V^fl/fl^MMTV^Cre^ mice measured by atomic force microscopy. Multiple peaks demonstrates characteristic multimodal stiffness profile of heterogeneous tumor breast tissue.

**Fig. S4:** C**ollagen profile of tumors and expression analysis of laminin in normal breast tissue**. (A) Representative images of Mason-Trichrome staining of total collagen content in breast tumor tissues from a2V^fl/fl^ or a2V^fl/fl^MMTV^Cre^ mice. The blue color shows staining for collagen protein and red color shows cytoplasm, scale bar 200µm. Graph shows quantification of collagen density. Values are presented as mean ± SE, n=5 each group, **P*<0.05. (B) Graph shows hydroxyproline amino acid content in breast tumor tissues from a2V^fl/fl^ or a2V^fl/fl^MMTV^Cre^ mice measured by hydroxyproline assay. Values are presented as mean ± SE, n=5 each group, *P<0.05. (C) Immunofluorescence analysis of laminin protein in tissue sections of normal breast from a2V^fl/fl^ and a2V^fl/fl^MMTV^Cre^ mice. The red color shows positive staining for laminins and blue color is DAPI staining for the nucleus. Scale bar 200µm. Graph shows mean intensities values. Values are presented as mean ± SE, n=7 each group, *P<0.05.

**Fig. S5: (A)** Western blot showing Giantin protein (~367kd) expression in protein lysates prepared from purified mammary epithelial cells from breast tissues of a2V^fl/fl^ or a2V^fl/fl^MMTV^Cre^ mice. Protein concentrations were normalized using β-actin (45kd). **(B)** SNA lectin staining demonstrating glycosylation in breast tissues from a2V^fl/fl^ or a2V^fl/fl^MMTV^Cre^ mice. The lectin staining appears as dark blue/brown color, magnification 10X, scale bar 200µm.

**Fig. S6: siRNA mediated knockdown of a2V.** HMEpC cells were transfected with three independent anti-a2V siRNA. A scramble siRNA (Scr) was used as a control. (A) Relative gene expression levels of a2V over endogenous GAPDH gene is shown. RNA interference assay was run in triplicate and repeated in at least three independent experiments. Values are shown in mean±SE, * P>0.05, n=3 for each siRNA. (B) Western blot showing a2V protein (55kd) expression in protein lysates prepared from HMEpC cells treated with Scr or siRNA-1. Protein concentrations were normalized using GAPDH (37kd).

**Fig. S7: Evaluation of a2V expression and collagen protein in human breast tissue:** Representative images of H&E, a2V and Mason-Trichrome staining in paired normal breast and primary tumor tissue from breast cancer patients reported with lymph node metastasis (LNM) of no LNM of tumors (n=5). Scale bar 500µm.
